# Deep Learning-Based Modified YOLACT Algorithm on Magnetic Resonance Imaging Images for Screening Common and Difficult Samples of Breast Cancer

**DOI:** 10.3390/diagnostics13091582

**Published:** 2023-04-28

**Authors:** Wei Wang, Yisong Wang

**Affiliations:** 1 College of Computer Science and Technology, Guizhou University, Guiyang 550001, China; 2 Institute for Artificial Intelligence, Guizhou University, Guiyang 550001, China; 3 Guizhou Provincial People’s Hospital, Guiyang 550001, China

**Keywords:** breast cancer, magnetic resonance imaging, artificial intelligence, deep learning technology, YOLACT algorithm model, deep convolutional neural network, modified YOLACT algorithm model, classification diagnosis

## Abstract

Computer-aided methods have been extensively applied for diagnosing breast lesions with magnetic resonance imaging (MRI), but fully-automatic diagnosis using deep learning is rarely documented. Deep-learning-technology-based artificial intelligence (AI) was used in this work to classify and diagnose breast cancer based on MRI images. Breast cancer MRI images from the Rider Breast MRI public dataset were converted into processable joint photographic expert group (JPG) format images. The location and shape of the lesion area were labeled using the Labelme software. A difficult-sample mining mechanism was introduced to improve the performance of the YOLACT algorithm model as a modified YOLACT algorithm model. Diagnostic efficacy was compared with the Mask R-CNN algorithm model. The deep learning framework was based on PyTorch version 1.0. Four thousand and four hundred labeled data with corresponding lesions were labeled as normal samples, and 1600 images with blurred lesion areas as difficult samples. The modified YOLACT algorithm model achieved higher accuracy and better classification performance than the YOLACT model. The detection accuracy of the modified YOLACT algorithm model with the difficult-sample-mining mechanism is improved by nearly 3% for common and difficult sample images. Compared with Mask R-CNN, it is still faster in running speed, and the difference in recognition accuracy is not obvious. The modified YOLACT algorithm had a classification accuracy of 98.5% for the common sample test set and 93.6% for difficult samples. We constructed a modified YOLACT algorithm model, which is superior to the YOLACT algorithm model in diagnosis and classification accuracy.

## 1. Introduction

Breast cancer is one of the most common cancers in the world, with high morbidity and mortality rates, posing a serious threat to women’s lives and well-being [1]. In the past 20 years, the incidence of breast cancer has shown a rapid increase, with a trend towards younger age groups and an unpromising morbidity and mortality rate [2]. Therefore, early diagnosis and treatment of breast cancer are still crucial for the effective prevention and treatment of breast cancer and for reducing the mortality of breast cancer. Clinically, the common examination methods for diagnosing breast cancer include color Doppler ultrasound, breast mammography, computed tomography (CT), MRI, and other methods [3]. In addition, two test sequences are commonly used for dynamic enhanced MRI; the T1 weighted image (T1W1) can reflect the distribution of breast fat and glands well, and the T2 weighted image (T2W1) can identify subcutaneous fluid composition [4].

MRI has been increasingly recognized by clinicians for its role in diagnosing, treating, and prognostically evaluating breast cancer [5]. Numerous studies have shown that with pathology as the “gold standard”, the sensitivity, specificity, and accuracy of MRI for breast cancer diagnosis are significantly higher than those of color Doppler ultrasound, mammography, CT, and other examination methods [6,7,8]. However, due to the obvious differences in the image characteristics of different molecular types of breast cancer, clinicians need to devote much energy and time to diagnosis combined with clinical characteristics, which cannot quickly and accurately confirm the condition and reduce the clinical treatment rate [9]. Therefore, computer-aided diagnosis can improve diagnostic efficiency, help patients to be treated quickly, and predict the disease more objectively and accurately, which is work of real value and wide application prospects [10].

Deep learning has emerged in computer vision and image processing, providing a new idea and way for computer-aided diagnosis [11]. With the continuous development of computer technology, the automatic analysis of medical imaging images with computer technology has become a research hotspot, a typical computer-aided diagnosis (CAD) method [12]. The ultimate goal of CAD is the automatic identification and classification of medical images to assist doctors in diagnosis. More and more hospitals have begun establishing CAD systems [13,14]. As one of the important missions of computer vision analysis, image classification has always been a popular research direction. The outstanding performance of the convolutional neural network (CNN) in image classification made it develop rapidly. The CNN model can automatically extract image features, automatically select image features, and conduct image classification without manual feature extraction and without being influenced by subjective factors, which can better assist doctors in diagnosis [15,16]. CNN is a commonly used deep learning network in clinical practice, primarily employed to identify and analyze both pathological and normal imaging data. CNN has tremendous potential in various clinical tasks such as segmentation, abnormal examination, disease classification, and diagnosis [17].

Deep learning has been successfully applied in computer vision analysis and has made great progress in medical image classification and analysis [18]. The detection and segmentation of breast cancer lesions are the prerequisites for identifying benign and malignant breast tumors and molecular subtypes [19]. The in-depth application of deep learning has improved the ability of MRI to diagnose and identify subtypes in breast cancer. Deep learning is prominent in image segmentation, first by extracting radiomic features from breast MRI, then training to identify benign and malignant breast tumors and molecular subtypes using supervised and unsupervised classical machine learning algorithms [20,21].

At present, large-scale breast MRI data sets are still limited. Therefore, it is urgent to establish a breast MRI data set with a large data volume, rich image information, and professional marker information to provide data support for training in lesion segmentation, benign and malignant tumor classification, and subtype identification in professional fields [22]. To quickly diagnose and extract the characteristics of each subtype of breast cancer and analyze them, our study used deep learning algorithms to construct an improved CNN algorithm model by obtaining MRI image data sets of benign and malignant breast tumors in an online database.

## 2. Methods

### 2.1. The Mask R-CNN Algorithm Model

**Figure 1:**
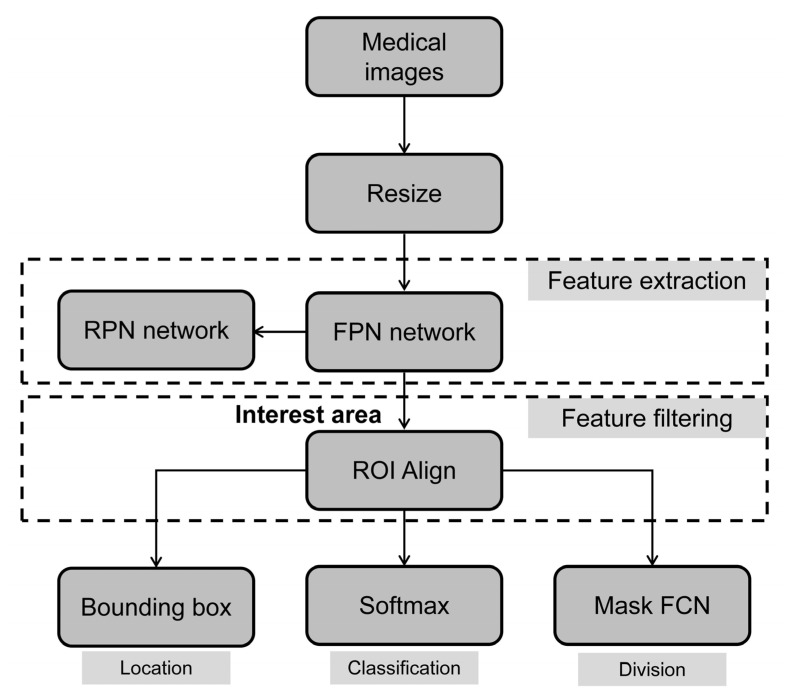
Schematic graph of the model structure of the Mask R-CNN algorithm.

The Mask R-CNN algorithm model is an improved version of the Faster R-CNN algorithm model. By improving and introducing a layer of fully convolutional segmentation branches, a model combining target detection and image segmentation was obtained, which can efficiently detect targets in the image and generate high-quality segmentation results for each target [23]. The Mask R-CNN algorithm model flow is shown in Figure 1.

The main body of the network model for the Mask R-CNN algorithm was based on Faster R-CNN, with the addition of a fully convolutional network to predict the semantic segmentation [24]. First, the residual network (Res-Net) was used as the feature to extract the skeleton network, combined with a feature pyramid network (FPN) to utilize better high-level semantic features and low-level texture features that extract multi-scale information in the image [25].The bilinear interpolation method was applied to the original region of interest (ROI) pooling to address the issue of the candidate box extraction process sampling an integer value for the tensor’s sampling point [26]. As a result, the region of interest could be completely aligned with the corresponding feature region in the original image. Retaining the fractional portion of the boundary tensor of the region-of-interest solved the mismatch between the candidate box of the ROI pooling filter and the original target, thereby enhancing the accuracy of candidate box detection results [27].

In the Mask R-CNN image processing workflow, the residual network was used as the feature extraction network to generate a multi-scale feature map from the preprocessed input image [28]. The FPN sampled the feature maps at different scales. The top-down sampling path was a nearest-neighbor up-sampling alternative from the highest layer, which was easier to operate, on the one hand, and reduced the training parameters, on the other hand. The horizontal connection was to fuse the up-sampled feature map and the feature map of the same size generated from the bottom to the top. Then, a 3 × 3 convolution was performed on the fused features to eliminate the image aliasing effect. The results were input into the region proposal network (RPN), and the horizontal–vertical ratios of three different scales were used to generate anchors of different sizes on each pixel. According to the image intersection over union, the corresponding ratio was obtained by comparing the prediction box and the real box repetition rates. Subsequently, it was fed into the region-of-interest align (ROI Align) together with the feature map. In this layer, MaskR-CNN replaced the ROI Pooling layer in Faster R-CNN with ROI Align. As a result, the target position information in the ROI Align process was more clearly calibrated, without using the rounding method on the feature map. Finally, the detection, localization, and segmentation loss functions were calculated separately, and the high-quality target segmentation results mapped by the target detection were generated simultaneously.

### 2.2. The YOLACT Algorithm Model

In the network structure of the YOLACT algorithm model, the feature pooling operation was removed, and the whole task was divided into two parallel subtasks, namely the prototype network branch and the target detection branch [29]. The formula used in the YOLACT algorithm model is shown as fellow.

(1) $${N\mathrm{um}}_{achor}=4+c+k$$

(2) $$M=\sigma ({PC}_{T})$$

(3) $$L(o,p)=L_{IoU}(o)+L_{score}(p)$$

(4) Lscore=1−p

(5) $$L_{IoU}=\frac{1}{1+e_{k}\times (o-0.5)}$$

(6) $$o=\frac{area(C)\cap area(G)}{area(C)\cup area(G)}$$

(7) $$P=\frac{TP}{TP+FP}$$

(8) $$R=\frac{TP}{TP+FN}$$

(9) $$AP=\sum_{r=0}(R_{n+1}-R_{n})\rho \mathrm{int}erp(R_{n+1})$$

**Figure 2:**
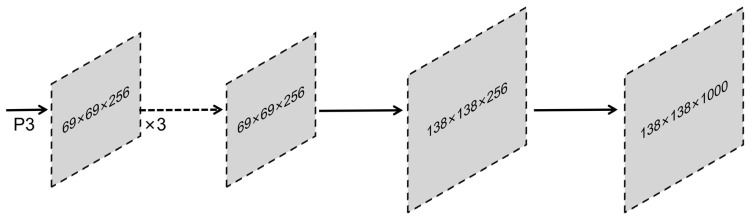
Schematic illustration of the structure of prototype network.

**Figure 3:**
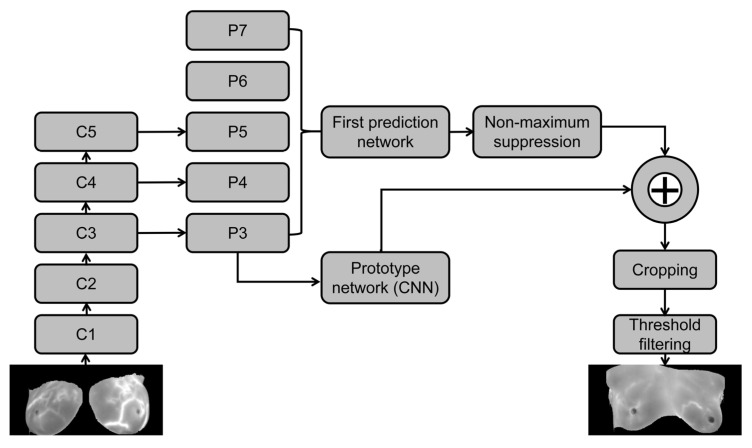
Schematic illustration of the structure of the YOLACT algorithm model.

1. Prototype network branch: the network structure of FCN was used to generate the prototype mask, as shown in Figure 2. The feature mapping P3 generated by the feature pyramid network structure passed through a set of FCN network structures, first through a layer of 3 × 3 convolution, then a layer of 1 × 1 convolution, followed by up-sampling to generate k prototypes of size 138 × 138, in which k was the mask coefficient.
2. Target detection branch: This branch predicted the masking coefficient for each anchor. As shown in Formula (1), 4 of them represent the candidate box information, the “c” represents the category coefficient, and “k” is the masking coefficient generated by the prototype network. Through the linear operation of the mask branch and the prototype mask, the predicted target’s location information and mask information could be determined by combining the results of the two branches. Finally, linear addition and multiplication operations were performed with the prototype mask after generating the corresponding mask coefficients for all targets. Then, clipping was performed according to the candidate box. Finally, the category was subjected to threshold filtering. That is, each target’s corresponding mask information and position information was obtained. The specific calculation is shown in Formula (2). P is the set of prototype masks obtained by multiplying the length and width of feature mapping and the masking coefficient. C represents the product of the number of instances passing through the network and the masking coefficient. The σ is the sigmoid function, and M is the combined result of the prototype mask and the detection branch.
3. Module generalization: The model’s prototype generation and mask coefficient can be added to the existing detection network. The flowchart of the YOLACT algorithm model is shown in Figure 3.

### 2.3. The Modified YOLACT Algorithm Model

In practical application, the pre-training network model often cannot handle the corresponding tasks directly. For example, in the image classification task, the Rider Breast MRI public data set has 80 categories and often fewer categories for the small-scale data set, so it is necessary to fine-tune based on the network model parameters, such as adjusting the number of categories in the classification layer, establishing a network model suitable for the actual task, and formulating strategies for specific tasks faster. Our study modified some training parameters of the original model and introduced the difficult-sample mining mechanism to improve the model’s performance. As shown in Formula (3), L_IoU_ and L_score_ represent position and category errors, respectively.

Total confidence 1 is subtracted by the classification confidence *p*, and the result represents the classification error value L_score,_ where p is the probability value output by the classification layer. The calculation process is shown in Formula (4). L_IoU_ is the target position error, and the calculation process is shown in Formulas (5) and (6).

Formula (6) calculates o as the intersection over union (IoU), which is derived from the ratio of the intersection and union of the real area and the predicted area, and initially sets the threshold to 0.5 to determine whether the candidate box is the target domain. In Formula (5), the difference value between the two is taken as the main discrimination index. To enhance the judgment criteria of challenging samples and make inter-sample errors affecting detection outcomes more apparent, we introduce the sensitivity coefficient k.

**Figure 4:**
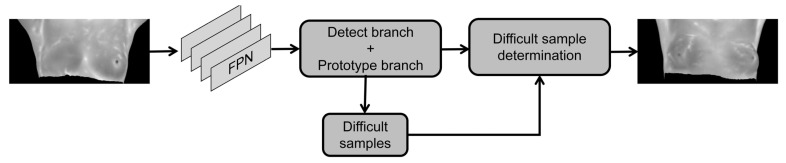
Flow chart of the introduction of the difficult-sample mining mechanism.

In Formula (3), L (o, p) is the final evaluation result derived from the algebraic sum of the sample category and position error. The threshold interval is set to determine whether the test sample is a difficult sample or not. The specific process is shown in Figure 4. The generated sample is judged after the mask is synthesized. Suppose the error value is in the set interval. In that case, the sample is judged as a difficult sample and returned to the detection branch and the prototype branch after the feature extraction network to solve the problem of model overfitting and insufficient data volume and improve the model detection accuracy.

### 2.4. Database Construction and Data Preprocessing

**Figure 5:**
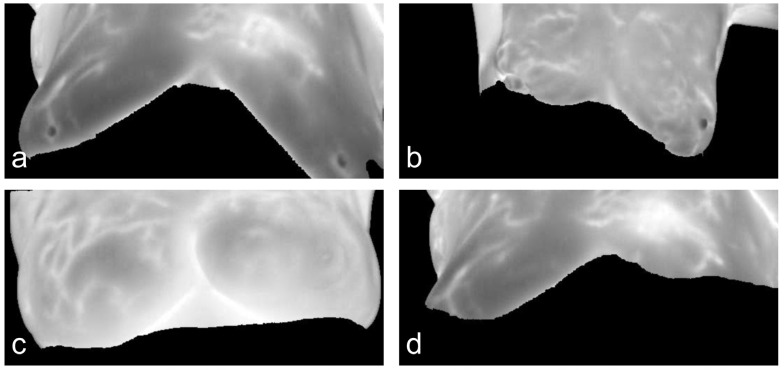
Representative MRI images of breast cancer patients. (**a**) A 48-year-old female patient with right breast cancer; (**b**) A 57-year-old female patient with bilateral breast cancer; (**c**) A 61-year-old female patient with right breast cancer; (**d**) A 55-year-old female patient with bilateral breast cancer.

**Figure 6:**
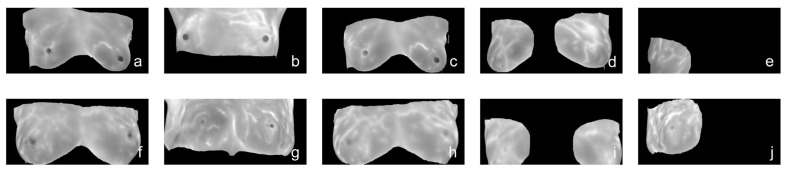
Preprocessing of normal breast samples and breast cancer MRI images, including the image of normal breast samples (**a**), image rotated 180 degrees (**b**), horizontal mirror image (**c**), cropped original image (**d**), and lesion image (**e**); image of breast cancer samples (**f**), image rotated 180 degrees (**g**), horizontal mirror image (**h**), cropped original image (**i**), and lesion image (**j**).

Breast cancer MRI images were obtained from the Rider Breast MRI public dataset (https://www.cancerimagingarchive.net/, accessed on 20 December 2022), and the data type was breast magnetic resonance imaging (MRI) [30]. Among them, 2400 DCM format data pieces of 288 × 288 size were selected as the experimental training set. The selected data were converted into processable JPG format images. The model was geometrically transformed (rotated, mirrored, cropped, scaled) to expand the data to 6000 different data pieces with difficult positive samples for model testing. Some MRI image data of experimental breast cancer patients is shown in Figure 5. Part of the preprocessed datasets is shown in Figure 6, in which some of the data contain the lesion area corresponding to the global image.

**Figure 7:**
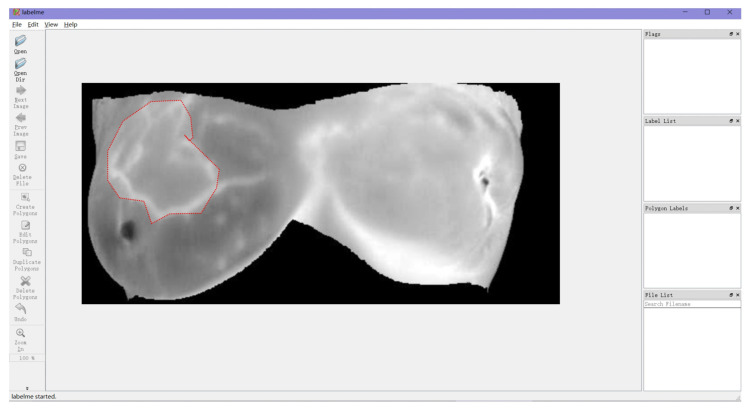
Breast cancer lesion labeled with Labelme software. Note: the red area indicates the range of the cancerous lesion in the breast.

The lesion location and shape in the original image of the Rider Breast MRI public data sets were labeled according to the lesion area location using the Labelme software (https://sourceforge.net/projects/labelme/, accessed on 20 February 2023). A diagram of the Labelme-software-labeled breast cancer lesion areas is shown in Figure 7. The labeled data consisted of 4400 normal samples with corresponding lesion labels and 1600 images of difficult samples. All the labeled data were randomly grouped in a certain proportion, of which 4800 samples were used as the training set and the remaining 1200 as the test set.

**Figure 8:**
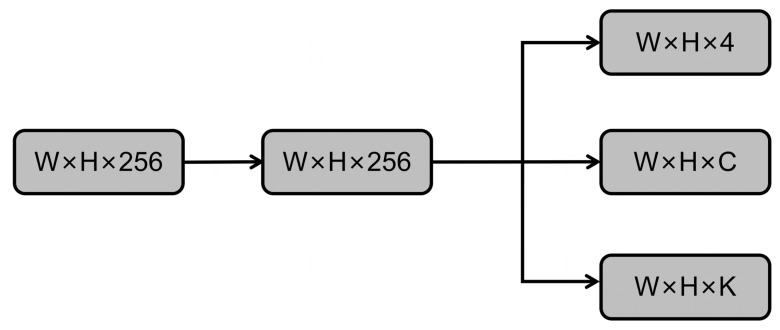
The YOLACT algorithm model predicts the network structure of breast cancer.

In consideration of both speed and performance, YOLACT employs a ResNet-101 network as the backbone detection network, adopting FPN as in the case of RetinaNet. The head structure of the prediction network was designed as shown in Figure 8, and the three layers generated on the right side represent four position information values, C values representing category information, and k mask coefficient values.

**Figure 9:**
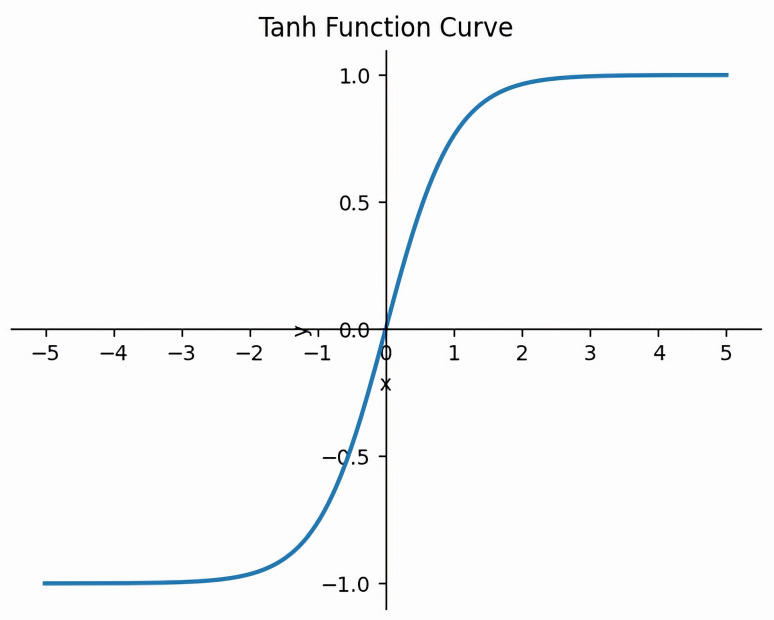
Tanh function curves of breast cancer MRI images predicted by the YOLACT network model.

In the training process of the YOLACT network model, the front-end convolutional layer retains the original feature pyramid structure, to ensure that the prototype mask in the original network structure is easily removed from the final mask and the Tanh activation function with function interval [−1, 1] continues to be selected. The function image is shown in Figure 9.

## 3. Results

### 3.1. Data Analysis Environment Construction and Test Results

In the pre-training parameter setting, according to the initialization weight of the pre-training network, the user-defined score threshold was set to 0.5. The number of iterations was initially set to 10,000. The decision to continue the breakpoint retraining was made according to the test results, to converge the loss value further. Batch parameters were initially set to four, according to the experimental graphic card model, to prevent video memory overflow, and the step length coefficient and Padding were kept at 1. The parameters were set as shown in Table S1.

**Figure 10:**
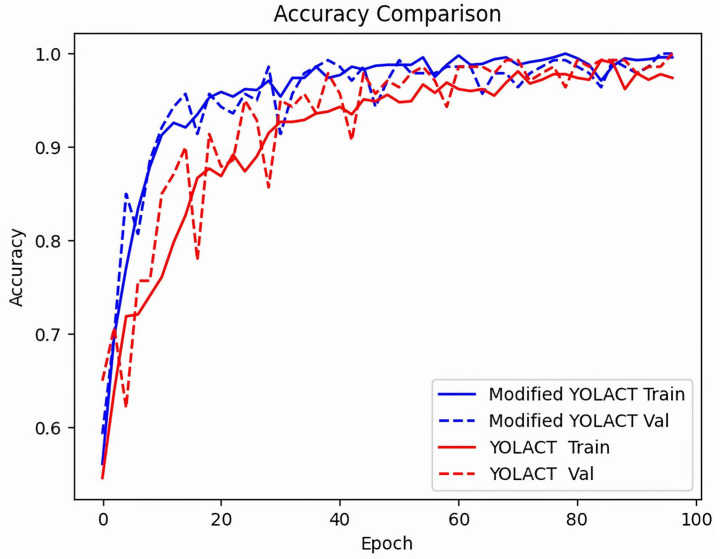
Results of PyTorch program operation.

The experimental environment was built based on the CUDA10 parallel computing platform and CUDNN7.3 deep neural network GPU acceleration library. The programming language used to write related functional modules was Python. The deep learning framework was based on PyTorch 1.0 version, and the GPU was configured as NVIDIARTX2080TI with 11 g of memory. The detection results of the program operation are shown in Figure 10.

**Figure 11:**
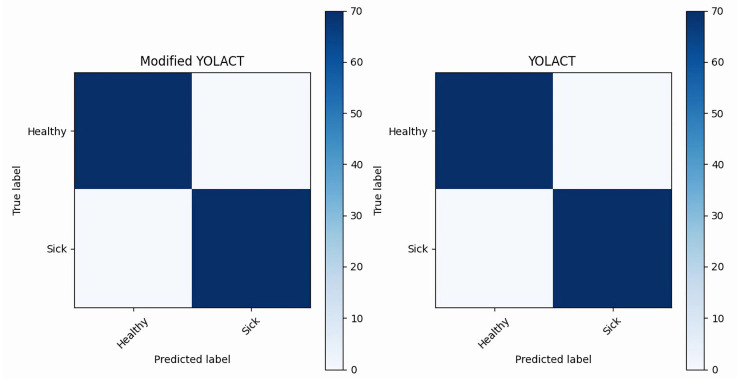
Diagnostic accuracy analysis of the YOLACT and modified YOLACT algorithm models for the training and test sets.

As shown in Figure 11, the training accuracy and test accuracy of YOLACT and modified YOLACT algorithm models change gradually with the increase in training times; the training accuracy of the model becomes higher, and the test accuracy also becomes higher, while the modified YOLACT algorithm model can achieve higher accuracy. This result shows that the modified model has better classification performance.

**Figure 12:**
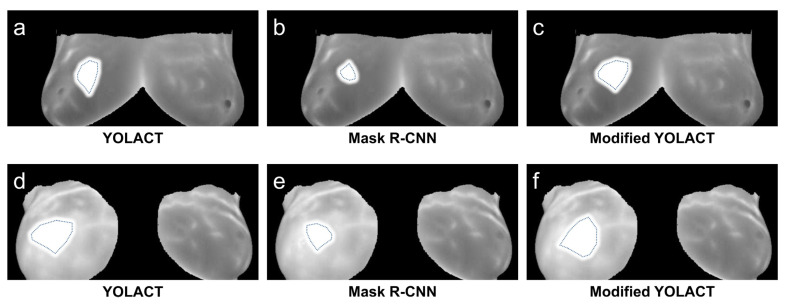
Confusion matrix of the type classification results of the modified YOLACT algorithm model. (**a**,**d**) YOLACT algorithm model marks the lesion area; (**b**,**e**) Mask R-CNN algorithm model marks the lesion area; (**c**,**f**) Modified YOLACT algorithm model marks the lesion area.

The resulting confusion matrix for classifying the test sets of common and difficult samples separately using the trained modified YOLACT algorithm model is shown in Figure 12. The ordinate of the confusion matrix represents the real labels, and the abscissa represents the predictive labels of the model. In general, the darker the color of the main diagonal of the confusion matrix, the higher the classification accuracy. According to the confusion matrix, the classification accuracy of the modified YOLACT algorithm model is 98.5% for the common sample test set and 93.6% for the difficult samples.

### 3.2. Comparison of the Diagnostic Accuracy among the Three Algorithmic Models for the MRI Images of Breast Cancer

The mean average precision (mAP) is used as the criterion for detecting image data analysis results. As shown in Formulas (7) and (8), the precision rate (P) indicates the total set ratio of the common samples to the difficult samples in the test results predicted by the model. The recall rate (R) represents the total set ratio of the common samples to the missed samples in the test results. In Formula (9), average precision (AP) is the mean of the maximum precision rate under different recall rates. All the means obtained are averaged to obtain the final mAP.

Table S2 compares the results of the YOLACT algorithm model, the Mask R-CNN algorithm model, and the modified YOLACT algorithm model. The data analysis results show that compared with the YOLACT algorithm model, the modified YOLACT algorithm model with the introduction of a difficult-sample mining mechanism enhances the detection accuracy of the common sample images and difficult samples by nearly 3%. However, compared with Mask R-CNN, it is still faster in running speed, and the difference in recognition accuracy could be clearer.

**Figure 13:**
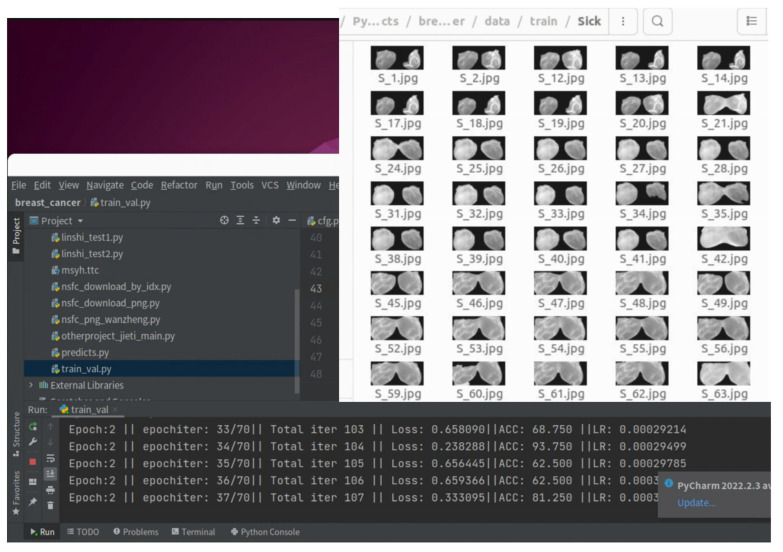
Detection results compared to YOLACT, Mask R-CNN, and modified YOLACT algorithm models.

As shown in Figure 13, the results of different algorithmic models were compared. All three algorithm models can distinguish whether the detected image is a breast cancer image in most breast cancer MRI image data. The modified YOLACT algorithm model used in this study exhibits a higher detection accuracy for images containing lesion areas. In addition, the segmentation result is closer to the mark in the original data set, which can better detect the breast cancer lesion area and segment the texture.

## 4. Conclusions

Among many imaging techniques, MRI is widely used in the clinical diagnosis of breast cancer due to its advantages of high resolution and no damage. It assists radiologists in diagnosis and decision making, which greatly improves clinicians’ diagnosis and treatment efficiency [3,6,31]. The concept of deep learning was first proposed by Hinton et al. in 2006 [32]. As an emerging technology in machine learning algorithms, its motivation is to establish a neural network that simulates the human brain for analysis and learning. Its essence is to layer the observation data further to abstract low-level features into high-level feature representations. The rise and prominence of deep learning and its successful application in image recognition, segmentation, labeling, and other aspects challenge traditional machine learning strategies [33]. The application of deep learning in the classification and diagnosis of MRI images in breast cancer has also further improved the early diagnosis rate of breast cancer.

The U-Net algorithm is the most commonly used deep learning algorithm for breast cancer MRI image lesion segmentation. The improved 3DU-Net algorithm based on this algorithm can significantly improve the diagnostic accuracy of radiologists with sufficient training [34]. The YOLACT algorithm model in this study was improved through the breast cancer MRI image data in the Rider Breast MRI public data set for model training and the introduction of the difficult-sample mining mechanism for difficult sample images, such as unbalanced image samples and obscure image detection features. Then, the modified YOLACT algorithm model was constructed. It is found that compared with the YOLACT algorithm model and the Mask R-CNN algorithm model, the modified YOLACT algorithm model has higher accuracy and runs faster.

The main task of breast MRI image classification is to identify benign and malignant images based on the CNN algorithm [35]. In our study, the modified YOLACT algorithm model was used to detect the common sample images and difficult sample images in the test set with good adaptability, and the false-detection rate was significantly reduced, thus improving the performance of the YOLACT algorithm model in medical image detection and segmentation tasks. Furthermore, Truhn et al. studied the benign and malignant diagnosis of breast MRI images with artificial ANN network (ANN) and CNN algorithms. They found that despite the adjustment of the ANN method, the accuracy of CNN was still higher than that of ANN [36]. Although great success has been achieved in predicting benign and malignant breast tumors by extracting MRI features, these studies mainly rely on semi-automatic feature extraction methods. Still, there are far more traditional machine learning models than deep learning models.

Some researchers have proposed an enhancement method based on multifractal images combined with edge local contrast of the tissue lesion area to further differentiate between benign and malignant breast tumors [37]. In addition, some research teams used the secondary transfer learning method to build auxiliary diagnosis systems for breast MRI images. The detection performance improved compared to the mainstream algorithm model [31]. The deep-learning-based detection method can better obtain the multi-scale information of the target image. However, when the detection target feature is insignificant, it is prone to false and missed detection with non-target regions containing similar features [38]. Our study introduced the difficult-sample mining method based on the YOLACT algorithm model for unbalanced image samples and insufficient image feature information. The dataset utilized for the model training was the Rider Breast MRI dataset of breast cancer. It is found that compared with the YOLACT algorithm model, the detection accuracy of the common sample image and the difficult sample image of the modified YOLACT algorithm model with the difficult-sample mining mechanism was improved by nearly 3%. Compared with Mask R-CNN, it is still faster in running speed, and the difference in recognition accuracy is not obvious.

With the rapid development of artificial intelligence, computer vision, and other related fields, deep learning has also been applied in medical image classification and detection and has achieved remarkable results [39]. Deep learning is becoming a leading machine learning tool in general imaging and computer vision. With its revolutionary breakthroughs in the exploration and application of machine vision and natural language processing and its potential in supplementing image interpretation, enhancing image representation and classification, it can also be widely used in medical image processing [40,41]. CNN uses local connection and weight sharing to take images directly as the network input, avoiding the cumbersome process of feature extraction and data reconstruction in the traditional recognition algorithm, enhancing its migration ability, and having great advantages in the processing of medical images [42].

In conclusion, a modified algorithm based on the YOLACT algorithm model was constructed in this study. The modified YOLACT algorithm model was superior to the YOLACT algorithm model. The classification accuracy of the modified YOLACT algorithm model for common sample test sets was 98.5%, and the classification accuracy for difficult sample test sets was 93.6%. The modified YOLACT algorithm model improved the diagnostic efficiency of common samples in breast cancer MRI images, showing a good ability to distinguish difficult sample images. Although this study has achieved some results in the design and performance evaluation of the modified YOLACT algorithm model, there are also the following limitations in this study. First, the MRI images of breast cancer used in this study are from the Rider Breast MRI public data set. Although these datasets are recognized, the limitations of data sources may lead to a reduction in the generalization ability of the algorithm. Future research should consider using more sources of data to improve the stability and generalization ability of the model. Secondly, this study compares the modified YOLACT algorithm model with Mask R-CNN. Although these comparisons can demonstrate the advantages of the modified YOLACT algorithm model, they are limited to these two algorithms. Future research should consider comparing the modified YOLACT algorithm model with other advanced algorithms in order to evaluate their performance more comprehensively. Finally, this study did not compare the results of the modified YOLACT algorithm model with histology (the gold standard for tumor diagnosis). Future research should compare the diagnostic results of the algorithm with histological results in order to more accurately assess the diagnostic accuracy of the algorithm. In conclusion, the goal of this study is to classify and diagnose MRI images of difficult breast cancer, but more factors may need to be considered in practical clinical applications, such as image differences caused by different MRI equipment and parameter settings, patient age, and medical history. Therefore, more validation and testing is needed before applying this research result to clinical practice.

## Data Availability

The article’s data will be shared on reasonable request to the corresponding author.

## Supplementary Materials

The following supporting information can be downloaded at: https://www.mdpi.com/article/10.3390/diagnostics13091582/s1, Table S1. Data analysis and parameter setting; Table S2. Quantitative analysis of the diagnostic accuracy of different algorithm models for MRI images of breast cancer.
