# Supplementary material for: Deep Learning-Based Modified YOLACT Algorithm on Magnetic Resonance Imaging Images for Screening Common and Difficult Samples of Breast Cancer
Source: Diagnostics (Basel). 2023 Apr 28;13(9):1582. doi: 10.3390/diagnostics13091582 (PMC10177566; doi:10.3390/diagnostics13091582)
Supplement: Supplementary file 1 [file diagnostics-13-01582-s001.zip › diagnostics-2266703-supplementary.pdf]

Table S1 Data analysis and parameter setting

Parameter name	Parameter value
Weight	Initial weight
Learning rate	0.0002
Iterations	10000
Batch processing capacity	4
Step	1
Padding	1

Table S2 Quantitative analysis of the diagnostic accuracy of different algorithm models for MRI images of breast cancer

Image type	Algorithm model	Average accuracy (%)	Average running time (s)
Routine samples	YOLACT	87.20%	3.5
	Mask R-CNN	81.30%	15
	Modified YOLACT	98.30%	4
Difficult samples	YOLACT	84.40%	4.5
	Mask R-CNN	79.40%	20
	Modified YOLACT	93.60%	5
